# Sustainable Valorization of Coffee Silverskin Waste: Pressurized Liquid Extraction of Bioactive Compounds

**DOI:** 10.3390/foods14040615

**Published:** 2025-02-12

**Authors:** Sokratis E. Koskinakis, Chrysanthos Stergiopoulos, Christoforos Vasileiou, Magdalini Krokida

**Affiliations:** Laboratory of Process Analysis and Design, School of Chemical Engineering, National Technical University of Athens, 9 Heroon Polytechniou St., Zografou Campus, 15780 Athens, Greece; chrisxp3@hotmail.com (C.S.); xristofvas@gmail.com (C.V.); mkrok@chemeng.ntua.gr (M.K.)

**Keywords:** coffee industry, by-product, silverskin, pressurize liquid extraction, response surface methodology

## Abstract

Coffee silverskin, a by-product of the coffee roasting industry, offers significant potential for valorization due to its bioactive compound content. This study optimized the Pressurized Liquid Extraction (PLE) process for recovering phenolic compounds and caffeine from coffee silverskin. A factorial design identified the temperature and ethanol concentration as the key parameters influencing extraction yield, antioxidant capacity (TEAC), total phenolic content (TPC) and caffeine and phenolic acid contents. These factors were further optimized using a central composite design (CCD) and Response Surface Methodology (RSM). The optimal extraction conditions—140 °C and 55% ethanol—achieved a balanced enhancement across all responses: 12.1% extraction yield, 65.3 mg TE/g dry extract for antioxidant capacity, 88.4 mg GAE/g dry extract for total phenolic content, 56.7 mg caffeine/g dry extract, and 10.6 mg chlorogenic acid equivalents/g dry extract for phenolic acid content. Compared to conventional methods, the optimized PLE demonstrated superior bioactive compound recovery while employing environmentally friendly solvents. This approach underscores PLE’s potential as a sustainable technology for valorizing agro-industrial by-products, contributing to both waste reduction and the development of antioxidant-rich products.

## 1. Introduction

Coffee has established its importance through the years as one of the most globally consumed beverages, playing a vital role in both cultural and economic societies. The growing demand for coffee has led to a consistent increase in production, with global coffee output reaching over 170 million 60 kg bags annually in recent years [1]. This growing tendency in the coffee industry not only results in the widespread availability of coffee but also in the generation of significant quantities of by-products, particularly silverskin. Silverskin is the thin husk that detaches from the raw coffee bean during the roasting process and represents 4.2% (*w*/*w*) of the coffee cherry [2]. As coffee production increases, so too does the volume of silverskin produced, often leading to its disposal or use in low-value applications. However, recent research has begun to uncover the potential of coffee silverskin as a valuable source of bioactive compounds, suggesting new opportunities for its utilization in various industries.

Coffee silverskin is particularly rich in phenolic compounds, which have garnered significant attention due to their potential as a natural antioxidant source. Among these phenolics, chlorogenic acids are notably abundant in silverskin and are recognized for their capacity to neutralize free radicals [3]. In addition, these compounds are crucial in preventing the onset of various chronic diseases, including cardiovascular diseases, diabetes, and cancer [4]. The robust antioxidant profile of coffee silverskin not only underscores its potential health benefits but also its suitability for applications where the preservation of oxidative stability is essential [5]. The ability of these antioxidants to mitigate oxidative processes positions coffee silverskin as a valuable natural source for enhancing the longevity and safety of various products, particularly in contexts where oxidative degradation is a concern. The antioxidant content in coffee is largely determined by its chlorogenic acid content rather than its origin or total phenolic and heavy metal contents [6].

Pressurized Liquid Extraction (PLE) is emerging as an efficient technology for extracting phenolic compounds from plant materials, including coffee silverskin. PLE operates under high pressure and temperature, maintaining solvents in a liquid state, which enhances the solubility of target compounds and accelerates the extraction process [7]. This technique allows for the use of water and other environmentally friendly solvents under subcritical conditions, therefore increasing the yield of phenolic compounds without degrading thermally sensitive components [8,9]. The use of PLE is particularly advantageous for extracting antioxidants like chlorogenic acids, as the extraction is performed in a close system under controlled temperature and thus prevents their degradation [10,11,12]. The environmentally friendly nature of PLE, combined with its effectiveness, positions it as a leading technology for the valorization of coffee silverskin.

Several studies have explored the extraction of bioactive compounds from coffee silverskin using different technologies, with ethanol and water being the most commonly used solvents. Ethanol is favored due to its efficacy in extracting a wide range of phenolic compounds [13,14]. It is also regarded as a safe and environmentally friendly solvent, suitable for applications in the food and nutraceutical industries [15]. Water has also proven effective in extracting phenolics from silverskin due to its ability to dissolve polar compounds at elevated temperatures [16]. The combination of ethanol and water, either in sequential or simultaneous extractions, has been shown to optimize the yield of phenolic compounds, making these solvents particularly attractive for green chemistry applications [17].

Various approaches have been investigated to extract bioactive compounds from coffee silverskin. Conventional solid–liquid extraction using ethanol and water remains widely used for its simplicity and ability to extract phenolics and antioxidants effectively [18]. Soxhlet extraction has also been employed, offering high-recovery yields by utilizing continuous solvent cycling at elevated temperatures [19]. Ultrasound-assisted extraction significantly enhances phenolic and antioxidant yields through the use of ultrasonic waves to improve mass transfer [20]. Microwave-assisted extraction reduces the extraction time while maintaining high bioactive yields by using microwave radiation to heat the solvent and sample rapidly [21]. Subcritical water extraction utilizes high-temperature water to extract phenolics efficiently, aligning well with environmentally friendly processing goals [22].

This study presents a novel application of Pressurized Liquid Extraction (PLE) to coffee silverskin, an underutilized agro-industrial by-product, aiming to unlock its potential as a source of high-value bioactive compounds. By employing a systematic approach using the design of experiments (response surface methodologies), the study optimizes key extraction parameters to maximize the recovery of total phenolic content, caffeine, and phenolic acids. Extractions were performed using water, ethanol, and their mixtures to evaluate the impact of solvent composition on the extraction efficiency. Statistical analysis through ANOVA validated the significance of the tested parameters and highlighted key interactions influencing the outcomes, guiding the refinement of the extraction conditions. Unlike conventional studies, this work not only focuses on the extraction but also provides a comprehensive characterization of the bioactives, linking their antioxidant activity to the optimized PLE conditions. Furthermore, the findings underscore the practical implications of upcycling coffee silverskin for applications in the food, nutraceutical, and cosmetic industries, aligning with the principles of the circular economy and sustainable resource management. This integrated and targeted approach distinguishes the study from previous works, offering both methodological innovation and practical relevance.

## 2. Materials and Methods

### 2.1. Coffee Beans

Coffee bean husks (2.46 ± 0.38 wt% humidity) of the Arabica variety (*Coffea arabica* L.) were used. They were kindly provided by the company Kafekopteia Loumidis, located in the city of Athens, Greece. The samples were ground using an electric knife mill Pulverisette 11 (Fritsch, Thessaloniki, Greece) and sieved until a particle size of 1 mm was achieved. The ground material was then stored in vacuum-sealed plastic bags at 4 °C until further extraction.

### 2.2. Chemical and Reagents

Ethanol (98% purity) and distilled water were used as extraction solvents, both sourced from Sigma-Aldrich (St. Louis, MO, USA). For the characterization of the extract, ultrapure water, methanol (HPLC grade), acetonitrile (HPLC grade), isopropanol (HPLC grade), Folin–Ciocalteau reagent, and sodium carbonate (Na_2_CO_3_) were used; these reagents were also obtained from Sigma-Aldrich. Other materials for characterizing the extract included 2,2-Diphenyl−1-(2,4,6-trinitrophenyl) hydrazyl (DPPH), 6-Hydroxy-2,5,7,8-tetramethylchromane-2-carboxylic acid (Trolox), 1,3,7-Trimethylxanthine (caffeine) 3,4,5-Trimethoxybenzoic acid (gallic acid), and 3-caffeoylquinic acid (chlorogenic acid), each with purity greater than 98%; these reagents were supplied by Extrasynthese S.A. (Genay Cedex, Genay, France).

### 2.3. Pressurized Extraction Process (PLE)

**Figure 1:**
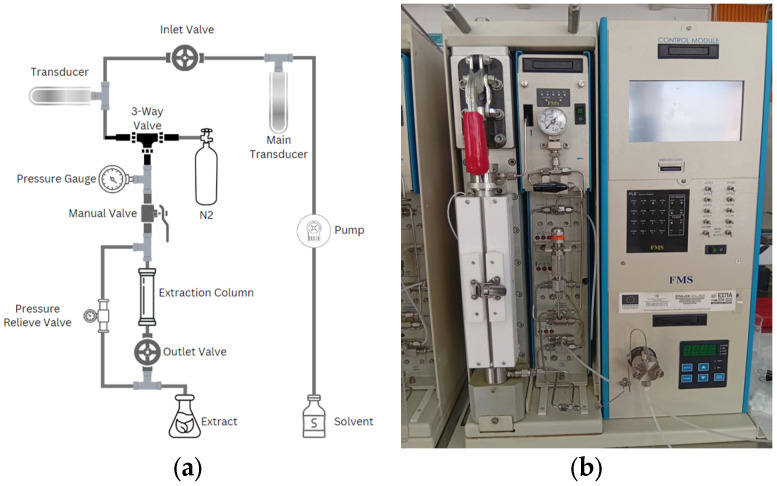
(**a**) Apparatus of the PLE. (**b**) PLE instrument (FMS inc).

The apparatus used for the experiments, as shown in Figure 1, was a PLE system (FMS, Fluid Management Systems, Inc., Waltham, MA, USA) comprising various components working in sequence to ensure efficient extraction. First, the solvent is pumped through the system using a high-pressure pump, entering through the inlet valve and regulated by a pressure transducer. The solvent is directed into a stainless-steel extraction column (100 mL volume, 20.6 mm diameter, and 30 cm length) via a 3-way valve. The extraction process begins by loading the column with the sample (range of 2–20 g raw material), followed by filling the column with solvent. The solvent is then heated by the heating blocks and kept at a constant pressure of approximately 120 bar to facilitate efficient extraction of the targeted compounds. The extraction proceeds in two stages: a static phase, in which the extraction column is pressurized and heated and the static time is manually regulated by the operator based on experimental requirements, followed by a dynamic phase. In the dynamic phase, fresh solvent is automatically pumped at a rate of 35 mL/min for 2.4 min, a parameter preset in the equipment for consistency across all experiments, to flush the extract into a glass container. Throughout the process, a pressure gauge continuously monitors the system, while a pressure relief valve ensures safety by preventing any excessive pressure buildup.

Following the extraction process, nitrogen gas (N_2_) is introduced into the system through the 3-way valve to flush out any remaining extract and solvent from the extraction column. The nitrogen flush ensures that all extract is transferred to the collector, minimizing losses and preventing any solvent residue from remaining in the column. The liquid extract is collected in a glass container and subsequently stored at approximately 4 °C to preserve its integrity.

### 2.4. Experimental Section

#### 2.4.1. Two-Level Factorial Design

A two-level factorial design was employed to efficiently assess the influence of five extraction-related parameters on five distinct responses, minimizing the number of experimental runs required. Using Design-Expert version 8 (StatEase, Inc., Minneapolis, MN, USA), eight experiments were performed in duplicate. This design, which excluded technical variables specific to the PLE instrument, strategically focused on extreme values of each parameter to swiftly identify the most influential factors. The investigated parameters included temperature, ethanol concentration in water, static extraction time, solid-to-solvent ratio, and number of extraction cycles. An extraction cycle is a repeated extraction performed on the same raw material under identical extraction conditions, following the initial extraction. The evaluated responses—extraction yield, antioxidant capacity, total phenolic compounds, caffeine content, and phenolic acid content—were analyzed using ANOVA. Pareto charts visually represented the statistical significance of each parameter’s influence, with t-values used to determine significance thresholds and Bonferroni limits applied to account for multiple comparisons. Parameters with t-values exceeding the Bonferroni-corrected threshold (t-value = 3.24) were deemed statistically significant, ensuring a rigorous and unbiased analysis. Although this 25-2 fractional factorial design provided an efficient means to screen influential parameters, it should be noted that it is a resolution III design, where the main effects are confounded with two-factor interactions. This means that significant results could stem from either individual factors or their interactions, which limits the ability to fully resolve these effects. Nonetheless, this approach remains valuable for the preliminary assessment and rapid identification of key parameters.

#### 2.4.2. Central Composite Design (CCD)

A CCD within the framework of response surface methodology (RSM) was employed to model the effects of the most impactful parameters—temperature and ethanol concentration—on five key responses: extraction yield, antioxidant capacity, total phenolic compounds, caffeine content, and phenolic acid content. Model equations were developed for each response to determine the relationships and interactions between the temperature and ethanol concentration. The significance of the variables, their interactions, and the overall model equations were evaluated using ANOVA, ensuring robust statistical validation.

The CCD, generated using Design-Expert version 8 (StatEase, Inc., Minneapolis, MN, USA), included 10 experiments with two central points (Table 1), each conducted in duplicate and considered separately. Non-essential parameters were held constant at resource-conserving levels to streamline the experimental process while maintaining focus on the critical factors. The static extraction time was fixed at 5 min, the solid-to-solvent ratio was maintained at 1:5, and the number of extraction cycles was limited to a single extraction to minimize resource consumption and ensure consistency across all experimental runs.

### 2.5. Obtaining and Characterizing the Extract

#### 2.5.1. Extraction Yield

To measure the extraction yield, the solvent system was removed using a rotary evaporator equipped with a V-800 vacuum controller (Buchi R-200 Rotavapor System, BUCHI Labortechnik AG, Flawil, Switzerland) equipped set at 100 mbar and a B-490 heating bath set to 60 °C. The residual material was then dried in a vacuum oven (Vacuum Drying Oven BOV-50V, Biobase Biodustry Shandong Co., Ltd., Jinan, China) for 3 h at 50 °C. The extraction yield (EY, %) was expressed as the percentage of the weight of the dry extracts (W_dry extract_) obtained relative to the weight of the dry sample (W_dry sample_) used for extraction through Equation (1):

(1) $$EY(\%)=\frac{W_{dry\;extract}(g)}{W_{dry\;sample}\;(g)}\cdot 100,$$

#### 2.5.2. Total Phenolic Content (TPC)

The TPC of the extracts was determined using the Folin–Ciocalteu method [23], which expresses total phenolics as gallic acid equivalents (GAE). For each measurement, 7.9 mL of deionized water, 0.1 mL of the sample (or deionized water for the blank), and 0.5 mL of Folin–Ciocalteu reagent were added to each test tube. Subsequently, 1.5 mL of saturated Na_2_CO_3_ solution was added sequentially to each test tube and the addition was held for a period of 30 s to 8 min. Tubes were then vortexed and left to react in a shaded place for 2 h. Absorbance was measured at 765 nm using a Bel Photonics M51 UV-Vis Spectrometer (Bel Engineering s.r.l., Monza, Italy). To quantify the total phenolic content, a calibration curve was constructed using gallic acid as the standard. The TPC of the extracts was calculated based on the calibration curve (R^2^ > 0.998) and expressed in terms of gallic acid equivalent (mg GAE/g dry leaves). The calibration curve used for expressing the TPC as GAEs is presented in Figure S1 in Supplementary Materials.

#### 2.5.3. Trolox Equivalent Antioxidant Capacity (TEAC)

The antioxidant capacity of the extracts was evaluated using the Trolox equivalent antioxidant capacity (TEAC) assay, with 2,2′-diphenyl-1-picrylhydrazyl (DPPH) as the radical and 6-hydroxy-2,5,7,8-tetramethylchroman-2-carboxylic acid (Trolox) as the antioxidant standard. The radical scavenging activity of the extracts against DPPH was measured spectrophotometrically at 515 nm after a 30 min reaction period. Absorbance measurements were performed with a Bel Photonics M51 UV-Vis Spectrometer (Bel Engineering s.r.l., Monza, Italy). The antioxidant capacity was expressed in Trolox equivalent (TE) using a calibration curve constructed with known concentrations of Trolox (R^2^ > 0.99), in the same manner as the TPC (mg TE/g dry leaves). The calibration curve used to translate the antioxidant capacity into TE is presented in Figure S2 in Supplementary Materials.

#### 2.5.4. Liquid Chromatography Analysis

The high-performance liquid chromatography with a diode-array detector (HPLC-DAD) method proposed by Psarrou et al. [24] (with some modifications) was used to detect the main compounds of the extracts. The HPLC apparatus consisted of a Prominence-i LC-2030 3D gradient pump and a diode array detector (Shimadzu, Kyoto, Japan). A Kromasil C18 column (5 µm, 250 × 4.6 mm, Nouryon AB, Göteborg, Sweden) was used under thermostated conditions at 30 °C. The samples were injected after filtration (0.45 μm, PVDF syringe filters, Teknokroma, Barcelona, Spain), and the flow rate was 1 mL/min. The solvent system consisted of water (A), methanol (B), acetonitrile (C), and isopropanol (D), with A, B, and C containing 0.2% trifluoroacetic acid. The initial composition of the mobile phase was 90% A, 6% B, 4% C, and 0% D. With linear gradients, the composition changed to 85% A, 9% B, 6% C, and 0% D within 5 min, 71% A, 17.4% B, 11.6% C, and 0% D within 30 min, and 0% A, 85% B, 15% C, and 0% D within 60 min. The injection volume was 20 µL, while the elution of compounds was monitored at 280 (caffeine) and 320 nm (phenolic acids). System control, data acquisition, and data processing were performed using the LabSolutions Workstation (Shimadzu, Kyoto, Japan). The identification of compounds was conducted using authentic reference standards for caffeine (CAF) and chlorogenic acid (CGA) by matching their retention times and UV spectra with those of the standards, as well as by comparing the results with the literature data. The quantification of compounds was based on calibration curves prepared using the corresponding authentic reference standards for caffeine and chlorogenic acid, with the rest of the phenolic acids detected expressed as chlorogenic acid equivalents (CGA eqs.), based on their characteristic UV spectra and data from the literature, according to the approximation used in literature [25,26]. All calibration curves were obtained in the range of 20–200 mg/L, analyzing four concentrations (20, 50, 100, and 200 mg/L) from duplicate samples. The detection of compounds was performed at the previously mentioned wavelengths and the produced linear equations presented with R^2^ > 0.998.

## 3. Results and Discussion

### 3.1. Two Factorial Design Results

Table 2 presents the results of a two-level factorial design to evaluate the influence of key parameters—temperature, ethanol concentration, static time, solid-to-solvent ratio (S/S), and number of extraction cycles—on the extraction performance from coffee silverskin. The responses examined include extraction yield (EY), Trolox equivalent antioxidant capacity (TEAC), total phenolic content (TPC), caffeine content, and phenolic acid content. This design enabled the identification of significant trends and interactions, providing critical insights into the role of the extraction conditions in optimizing bioactive compound recovery.

The results highlight the significant impact of temperature and solvent type on the extraction outcomes. The highest extraction yield (29.9%) was achieved at 190 °C using water as the solvent, a static time of 25 min, a 1:5 S/S, and no cycles. In contrast, the lowest yield (4.5%) occurred at 25 °C, with ethanol as the solvent, a 1:5 S/S, 5 min of extraction, and no cycles. This stark difference underscores the pivotal role of the temperature and solvent in facilitating cell wall disruption and enhancing solvent penetration, particularly under pressurized conditions. High temperatures enable water to act as an efficient extraction medium due to increased diffusivity and solubility, as corroborated by the study from Antony and Farid [27]. However, prolonged high-temperature exposure must be carefully managed to prevent the degradation of heat-sensitive compounds.

The antioxidant activity, measured as TEAC, peaked at 144.7 mg TE/g dry extract under conditions of 190 °C, ethanol as the solvent, a 1:50 S/S, a static time of 5 min, and two cycles. Conversely, the lowest TEAC (16.9 mg TE/g dry extract) was observed at 190 °C, with water as the solvent, 25 min of extraction, a 1:5 S/S, and no cycles. These findings indicate ethanol’s superior ability to extract antioxidant compounds, such as polyphenols, particularly at high temperatures. Ethanol’s selective solubility for polyphenolic antioxidants, combined with elevated temperatures, is well documented as a key factor in maximizing antioxidant activity [28].

Temperature played a dominant role in the extraction of total phenolic compounds. Higher TPC values were observed at elevated temperatures, likely due to the increased solubility and diffusivity of phenolics driven by enhanced kinetic energy and altered solvent polarity. This trend aligns with findings from Roselló-Soto et al. [29], emphasizing the importance of temperature in efficient phenolic compound extraction, while avoiding thermal degradation.

Caffeine extraction was influenced by both temperature and solvent type. The highest caffeine concentration (34.2 mg caffeine/g dry extract) was achieved using water at 25 °C, with a 1:5 S/S ratio and a static time of 5 min. Water’s higher affinity for caffeine compared to ethanol, combined with its effectiveness as a solvent, explains this result. The influence of temperature on caffeine extraction has been widely reported in the literature, with studies such as those by Nonappa and Kolehmainen [30] confirming its role in enhancing mass transfer and extraction efficiency. While the exact temperature conditions may vary, their findings align with the observed importance of solvent choice for optimizing caffeine recovery.

Phenolic acid content was maximized at 25 °C, with water as the solvent, a 1:5 S/S, a static time of 5 min, and two cycles. This suggests that solvent replenishment plays a critical role in facilitating phenolic acid extraction. However, higher temperatures also mildly improved phenolic acid recovery, likely due to enhanced solubility and cell disruption. These findings are consistent with recommendations in the literature for using mild conditions to preserve phenolic acid integrity while optimizing their extraction [31].

#### ANOVA Analysis of Two-Level Factorial Design Results

The two-level factorial design identified the primary parameters affecting the measured responses. Specifically, five parameters were included in the screening design: ethanol concentration in the solvent mixture, extraction temperature, static extraction time, solid-to-solvent ratio (S/S), and the number of extraction cycles. Low and high values for each parameter were determined based on the equipment’s capabilities and prior experience with PLE. The selected responses for evaluating the influence of these extraction parameters were the extraction yield, TEAC, TPC, caffeine content, and phenolic acid content. The experimental results for these responses are presented in Table 3. Statistical analysis was conducted using analysis of variance (ANOVA) to assess the significance of each parameter’s impact.

The ANOVA results in Table 3 indicate that temperature and ethanol concentration were the most significant parameters affecting extraction outcomes, as they had low *p*-values (<0.05) and high F-values. Temperature significantly influenced yield (F = 55.6, *p* < 0.0001), TEAC (F = 31.9, *p* = 0.0001), TPC (F = 583.8, *p* < 0.0001), caffeine (F = 9.44, *p* = 0.0097), and phenolic acids (F = 51.7, *p* < 0.0001), highlighting its role in enhancing solvent penetration and solute diffusivity. Ethanol concentration significantly impacted yield, TEAC, caffeine, and phenolic acids, emphasizing its selective solubility for bioactive compounds. Other parameters, such as static extraction time, solid-to-solvent ratio, and number of extraction cycles, had notable effects on specific responses, particularly for TEAC, TPC, and phenolic acid recovery.

**Figure 2:**
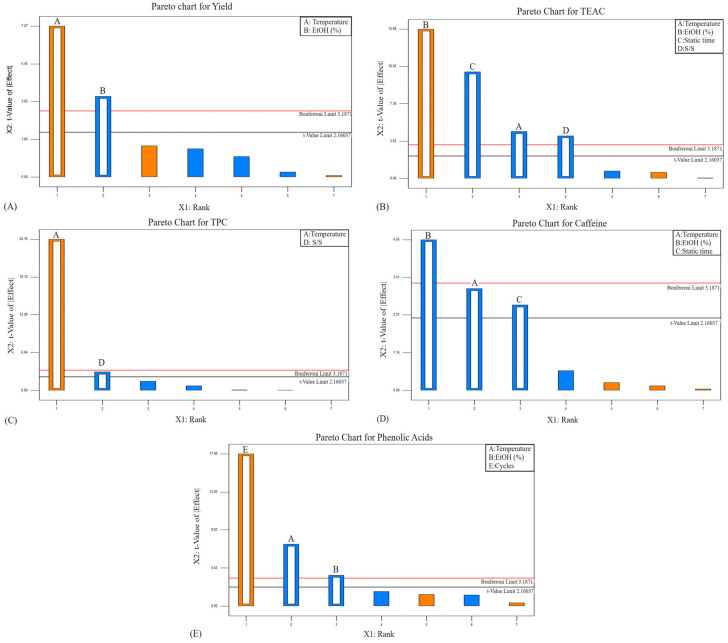
Pareto charts showing the effect size of extraction parameters on the measured responses. The letters above the rectangles represent A: Temperature, B: Ethanol concentration, C: Static time, D: solid-to-solvent ratio, and E: number of cycles. The bar heights indicate the influence of each parameter on (**A**) extraction yield, (**B**) TEAC, (**C**) TPC, (**D**) caffeine content, and (**E**) phenolic acid content.

The Pareto charts (Figure 2) highlight the relative impact of the extraction parameters on the measured responses, with bar lengths indicating effect magnitude and Bonferroni and t-value limits marking statistical significance thresholds. Parameters exceeding these thresholds are critical to the extraction process. For EY (Figure 2A), temperature and ethanol concentration are the most influential, enhancing solvent penetration and cell wall disruption. The TEAC (Figure 2B) is primarily driven by the ethanol concentration and static time, optimizing solubilization and bioactive recovery. The TPC (Figure 2C) is dominated by temperature, while the caffeine content (Figure 2D) depends on the ethanol concentration, temperature, and static time for improved solubility and mass transfer. In terms of the phenolic acid content (Figure 2E), the number of extraction cycles is most significant, alongside the temperature and ethanol concentration.

Overall, the Pareto charts confirm the temperature and ethanol concentration as primary variables across responses. These were further optimized using a CCD to enhance efficiency and accuracy, minimizing experimental runs while maximizing bioactive recovery.

### 3.2. CCD Results

Table 4 summarizes the results of the CCD, highlighting how each parameter influences the extraction responses. The data reveal a clear relationship between the temperature, ethanol concentration, and extraction outcomes. Higher values for both parameters generally enhance the extraction efficiency for specific compounds. As the temperature increases, the solvent’s polarity and solubility adjust, enabling more effective extraction of phenolic compounds due to changes in solvent behavior under high heat and high pressure. This improvement in physical properties likely facilitates the diffusion of phenolic compounds into the solvent, as elevated temperatures lower the activation energy required for desorption [32]. However, at temperatures exceeding 160 °C, all responses except for yield tend to decrease, reflecting the potential degradation of heat-sensitive compounds.

The addition of ethanol as a co-solvent enhances the extraction efficiency by improving solvent properties and enabling the recovery of both polar and non-polar compounds. Optimal conditions, such as 50% ethanol at 108 °C, achieved high caffeine content (68.1 mg/g) and TEAC (77.5 mg Trolox/g). Pure solvents (0% or 100% ethanol) were less effective, underscoring the importance of balanced hydroethanolic mixtures. Temperature also significantly impacted outcomes, with the highest yield (29.8%) observed at 190 °C and with 50% ethanol, though extreme temperatures risk degrading heat-sensitive compounds.

#### Statistical Models, Prediction, and Interpretation of Results

The Response Surface Methodology (RSM) was used to model the extraction process, focusing on temperature and ethanol concentration as key parameters. A central composite design (CCD) with five factor levels (±α, ±1, and 0) was implemented to ensure rotatability and consistent prediction variance. Quadratic models captured linear, interaction, and second-order effects, essential for addressing the nonlinear relationships between temperature, ethanol concentration, and extraction efficiency. The models were validated by ANOVA (Table 5), showing high F-values, low *p*-values, and strong R² values (0.90–0.96), confirming their predictive accuracy. These results highlight the importance of both linear and quadratic effects in optimizing extraction yield, antioxidant capacity, TPC, and caffeine and phenolic acid recovery.

The model for extraction yield demonstrated a strong fit, with an F-value of 83.9 and a *p*-value of less than 0.0001, indicating that the model is statistically significant. Both temperature and ethanol concentration exhibited significant linear and quadratic effects on the extraction yield. Equation (2) represents the prediction of extraction yield.

(2) $$EY\;(\%)=27.87304-\left(0.41033\cdot Temperature\right)–\left(0.11770\cdot Ethanol\right)+\;\left(2.25401\times 10^{-3}\cdot {Temperature}^{2}\right)+\left(1.13032\times 10^{-3}\cdot {Ethanol}^{2}\right),$$

**Figure 3:**
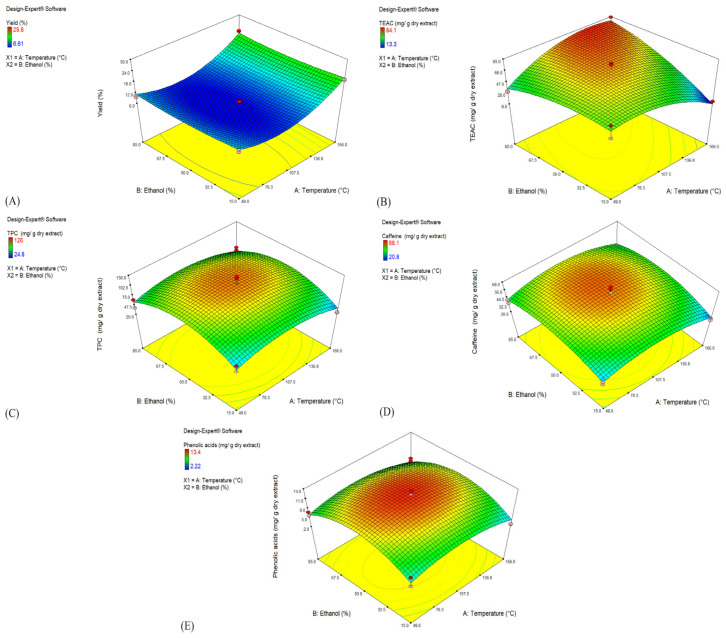
Response surface plot for: (**A**) extraction yield, (**B**) TEAC, (**C**) TPC, (**D**) caffeine, and (**E**) phenolic acids.

The trends in the response surface plot (Figure 3A) validate the predictions of the model. The yield increases with rising temperature, showcasing the strong influence of temperature on the extraction efficiency. At higher temperatures, solvent penetration is improved due to enhanced cell wall permeability and reduced activation energy for desorption, consistent with findings by Huamán-Castilla et al. [33]. At low temperatures, yields remain uniformly low regardless of the ethanol concentration, highlighting the need for sufficient thermal energy for efficient extraction. The ethanol concentration plays a secondary role, with intermediate levels (~50%) improving yields by balancing solvent polarity, optimizing the extraction of diverse compounds. The quadratic nature of the model underscores the importance of optimizing both parameters, as extreme values can decrease efficiency and compromise compound recovery.

The ANOVA results for TEAC reveal an F-value of 43.1, indicating a strong model fit. Significant contributions were observed from both temperature (*p* = 0.0011) and the interaction between temperature and ethanol concentration (*p* < 0.0001), highlighting their combined influence on antioxidant capacity. Additionally, the quadratic terms for both factors were significant, confirming the appropriateness of using a quadratic model to accurately capture the response behavior. The equation representing the model TEAC predictions is Equation (3).

(3) $$TEAC=30.05384+\left(0.63190\cdot Temperature\right)+\left(0.044557\cdot Ethanol\right)+\;\left(0.010710\cdot Temperature\cdot Ethanol\right)–(5.49075\times 10^{-3}\cdot {Temperature}^{2}-\;8.34984\times 10^{-3}\cdot 2{Ethanol}^{2}),$$

The response surface plot (Figure 3B) illustrates the combined effects of the temperature and ethanol concentration on TEAC. The trends show that higher temperatures enhance antioxidant recovery by improving solubility and reducing solvent polarity, while lower parameter values yield uniformly low TEAC, reflecting insufficient activation for antioxidant extraction. Optimal TEAC levels emerge within moderate ranges of both parameters, consistent with the findings by Kodchakorn and Kongtawelert [34]. The interaction of temperature and ethanol concentration amplifies these effects, highlighting the quadratic model’s utility in fine-tuning conditions for efficient antioxidant recovery while preserving compound integrity.

The TPC model exhibited high statistical significance, with an F-value of 33.9. Both the temperature and ethanol concentration were identified as crucial factors, along with their quadratic terms and interaction effects. This suggests a complex relationship between these parameters and the TPC that was effectively captured by the quadratic model. The equation representing the model of TPC predictions is Equation (4).

(4) $$TPC=-55.01350+\left(1.52489\cdot Temperature\right)+(3.42325\cdot \;Ethanol)–\left(7.38121\times 10^{-3}\cdot {Τemperature}^{2}\right)–\left(0.032850\cdot {Ethanol}^{2}\right),$$

The response surface plot (Figure 3C) highlights that the TPC increases with both the temperature and ethanol concentration, reaching an optimal peak at moderate levels of both parameters. This trend reflects the role of temperature in enhancing phenolic solubilization by reducing solvent viscosity and increasing molecular mobility, while the ethanol concentration optimizes solvent polarity for phenolic extraction. However, the TPC declines slightly at extreme values of these parameters, indicating phenolic sensitivity to high temperatures and excessive ethanol, which can cause degradation. This aligns with findings by Gunathilake et al. [35], who reported optimal TPC recovery at balanced conditions but observed declines when critical levels were exceeded.

The caffeine content and phenolic acid content models demonstrated high statistical significance, with F-values of 36.49 and 42.80, respectively. Both models indicated that the linear terms of temperature and ethanol concentration, as well as their quadratic components, significantly influenced the responses. The equations representing the model of the caffeine and phenolic acid content predictions are Equations (5) and (6).

(5) $$Caffeine=-19.81667+(0.89789\;\cdot \;Temperature)+(1.30453\cdot \;Ethanol)-(4.35271\times 10^{-3}\;\cdot \;{Temperature}^{2})-(0.011612\;\cdot \;{Ethanol}^{2}),$$

(6) $$\begin{array}{cc} Phenolic\;acids & =-5.80705+(0.16296\cdot Temperature)+(0.41178\\ & \cdot \;Ethanol)-(8.03335\times 10^{-4}\cdot {Temperature}^{2})\\ & -(4.05427\times 10^{-3}\cdot {Ethanol}^{2}), \end{array}$$

The response surface plot for caffeine (Figure 3D) shows that the caffeine content increases with rising temperature and ethanol concentration, reaching an optimal range at moderate levels of both parameters. However, at extreme ethanol concentrations or very high temperatures, the caffeine content declines. This trend is consistent with the findings from Jun et al. [36], where high hydrostatic pressure combined with ethanol significantly enhanced the caffeine extraction efficiency by improving solvent penetration and reducing processing time. The quadratic nature of the model highlights the importance of avoiding excessive heat or extreme ethanol levels to maintain caffeine integrity. For phenolic acids (Figure 3E), a similar trend was observed, with content peaking at moderate temperature and ethanol levels before declining under extreme conditions. These findings align with those of Cheaib et al. [37], who demonstrated that moderate ethanol concentrations stabilize phenolic compounds during extraction, while excessive heat leads to their thermal degradation. The role of ethanol in altering solvent polarity and facilitating the solubilization of phenolic acids is evident, while the quadratic terms in the model capture the decline in phenolic acids under non-optimal conditions.

The response surface plots for all the responses (Figure 2) consistently demonstrate that optimal extraction outcomes are achieved under moderate conditions of ethanol concentration and temperature. These optimal regions, located near the center of each response surface, highlight the delicate balance required to maximize the extraction efficiency. Elevated temperatures enhance solvent diffusion and cell wall permeability, while moderate ethanol concentrations reduce solvent polarity, enabling the recovery of a diverse range of bioactive compounds. However, the quadratic models also reveal that extreme values for either parameter can lead to suboptimal outcomes, such as the thermal degradation of heat-sensitive compounds or diminished solubility. The quadratic nature of the models proves essential for accurately representing the complex interactions between temperature and ethanol concentration that cannot be adequately captured by simpler linear models. Collectively, the results underscore the sensitivity of bioactive compound extraction to these parameters and demonstrate that only specific, well-defined ranges allow for maximal efficiency while preserving compound integrity.

### 3.3. Optimal Extraction Conditions and Model Validation

The models identified 140 °C and 55% ethanol as the optimal extraction conditions, balancing all five response variables (yield, TEAC, TPC, caffeine, and phenolic acids) for comprehensive extract quality. While the actual values (Table 6) were slightly lower than predicted, deviations remained within 15%, confirming the model’s reliability. This optimization strategy ensured a well-rounded bioactive profile, enhancing the extract’s applicability in various industries. Such an approach demonstrates the potential of PLE for delivering high-quality extracts tailored to diverse functional needs.

**Figure 4:**
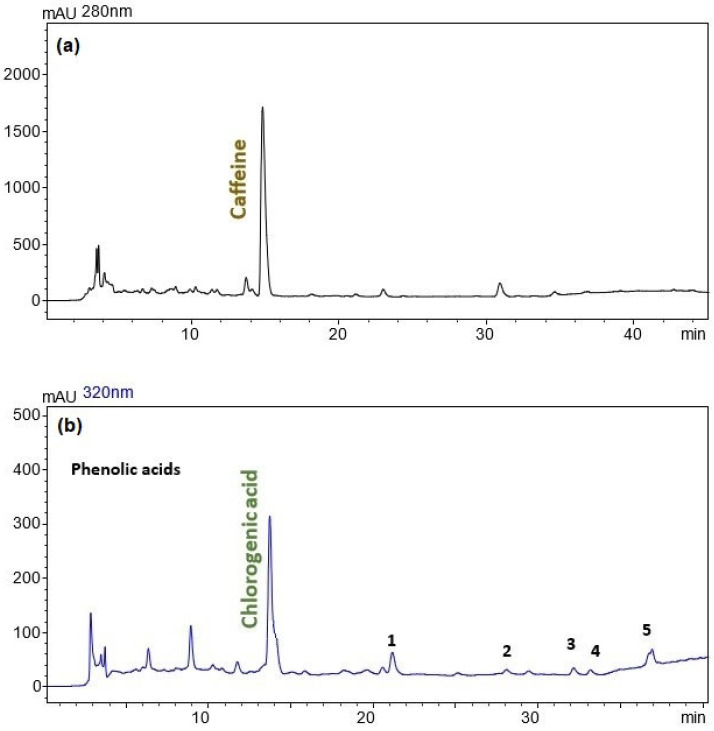
HPLC analysis of coffee silverskin extracts obtained under the optimized extraction conditions: (**a**) caffeine detection at 280 nm and (**b**) phenolic acid detection at 320 nm. Peaks 1–5 represent additional detected phenolic acids.

Figure 4 presents the HPLC-DAD analysis of the coffee silverskin extracts obtained under the optimized extraction conditions (140 °C and 55% ethanol). Figure 4a highlights the caffeine content detected at 280 nm, while panel 4b focuses on phenolic acids detected at 320 nm, with chlorogenic acid as the primary component.

The chromatogram at 280 nm (Figure 4a) shows a prominent caffeine peak at 14.516 min, confirming its substantial recovery under the optimized conditions. The high intensity of the caffeine peak aligns with the actual caffeine content (56.7 mg/g dry extract) reported in Table 6. This validates the effectiveness of the optimized PLE process in recovering caffeine, which is highly valued for its antioxidant and functional properties. The absence of significant additional peaks at this wavelength indicates the selectivity of the method for caffeine extraction.

The chromatogram at 320 nm (Figure 4b) highlights the presence of chlorogenic acid as the dominant phenolic acid at 13.208 min, along with minor peaks corresponding to other phenolic acids. The well-defined chlorogenic acid peak corroborates the phenolic acid content measured experimentally (10.6 mg CGA eq./g dry extract, Table 6). The observed distribution of phenolic acids reflects the ability of the PLE process to recover both major and minor phenolic compounds, ensuring a broad bioactive profile.

All UV spectra of the identified compounds, along with their retention times, are included in Table S1 in Supplementary Materials. The UV spectra of the identified compounds are consistent with those reported in the literature for caffeine and phenolic acids [38,39,40,41].

The HPLC results further support the reliability of the quadratic models and the chosen optimization strategy. The extraction conditions (140 °C, 55% ethanol) were selected to balance yield (12.1%), TEAC (65.3 mg TE/g dry extract), TPC (88.4 mg GAE/g dry extract), and caffeine and phenolic acid content, as shown in Table 6. Although the actual values for some responses, such as the TPC and TEAC, were slightly lower than the predicted values, the deviations remained within 15%, underscoring the robustness of the predictions.

A comparison with other extraction methods highlights the advantages of this optimized PLE approach. For example, Ginting et al. [22] utilized subcritical water extraction at 147.9 °C for 10 min with a 1:10 solid-to-solvent ratio, achieving a yield of 27.25%, TEAC of 13.72 mg/g dry extract, TPC of 51.86 mg GAE/g dry extract, and total phenolic acids of 2.70 mg/g dry extract. Castaldo [42] employed heating with stirring at 80 °C for 30 min with a 1:10 solid-to-solvent ratio, reporting TEAC values of 8.1 to 14.4 mg Trolox/g dry extract, TPC of 5.7 to 6.9 mg GAE/g raw material, caffeine concentrations of 27.7 to 34.3 mg/g dry extract, and total phenolic acids ranging from 8.77 to 17.15 mg/g dry extract. Wen [20] used an orbital shaker with ultrasonic pretreatment at 50 °C for 24 h and a 1:50 solid-to-solvent ratio with 80% methanol, achieving TEAC values of 3.51 to 7.08 mg Trolox/g raw material, TPC of 5.80 to 8.94 mg GAE/g raw material, and caffeine concentrations of 0.33 to 0.39 mg/g raw material. Similarly, Brzezińska et al. [43] used ultrasonic extraction at 60 °C for 30 min with a 1:45 solid-to-solvent ratio in 50% ethanol, yielding a TEAC of 25.43 mg Trolox/g dry extract, TPC of 8.9 mg GAE/g dry extract, caffeine content of 5.9 mg/g dry extract, and phenolic acids of 0.22 mg/g dry extract. Ballesteros [19] employed conventional extraction at 65 °C for 30 min with a 1:35 solid-to-solvent ratio in 60% ethanol, achieving a TEAC of 4.48 mg Trolox/g dry extract and TPC of 12.81 mg GAE/g dry extract. These comparisons underscore the strength of the optimized PLE process, which consistently achieves superior bioactive compound recovery across multiple responses.

It is crucial to acknowledge that the chemical composition of coffee silverskin, including its phenolic content and antioxidant properties, can vary depending on coffee variety, harvest conditions, and roasting methods. Studies have shown that Coffea robusta silverskin generally contains higher levels of phenolics and caffeine compared to *Coffea arabica*, resulting in stronger antioxidant capacity [44,45]. Additionally, post-harvest treatment and roasting intensity significantly influence the bioactive profiles of silverskin. For instance, dark-roasted arabica silverskin is often found to contain higher concentrations of certain chlorogenic acids compared to lighter roasts, thereby enhancing its antioxidant potential [46]. These factors emphasize the importance of considering the raw material characteristics when evaluating and applying extraction methods for optimal outcomes.

The optimized PLE process demonstrates clear advantages in terms of efficiency and environmental sustainability, but it is crucial to acknowledge its limitations in industrial-scale applications. One of the primary concerns is the high-pressure requirement, which exceeds 100 bar. This necessitates specialized equipment that increases not only the initial investment but also ongoing operational costs. The energy demands associated with maintaining high pressure and elevated temperatures are substantial, and without optimization, these factors can hinder the process’s economic feasibility. Furthermore, ethanol at a 55% concentration, while effective for extraction, poses flammability risks that require ATEX-compliant equipment, further complicating large-scale implementation and adding to the overall expense. Notably, advancements in ethanol recycling have demonstrated significant potential in reducing operational costs, with studies showing returns on investment as high as 133.5% under optimized industrial conditions [47]. This makes ethanol recycling an essential consideration for future implementations.

Another key issue is the scalability of PLE. Although it performs well under controlled laboratory conditions, its transition to industrial settings involves challenges related to safety, cost, and energy consumption. Compared to conventional extraction methods, PLE demands more stringent process control and higher energy input, which could limit its adoption in large-scale operations. While solvent recycling and energy-efficient systems could mitigate these drawbacks, they require additional infrastructure and further investment. Encouragingly, pilot-scale studies have demonstrated the feasibility of industrial-scale PLE, showcasing the ability to maintain product integrity and reduce waste [48]. Furthermore, the incorporation of green solvents like ethanol, recognized for their safety and minimal toxicity, underscores the method’s alignment with sustainability objectives [49].

Despite these challenges, PLE remains a promising method for selective extraction of thermolabile compounds. However, its industrial viability depends on future improvements in process design, such as reducing the pressure and temperature requirements or developing safer, lower-cost solvent alternatives. Addressing these aspects will be critical in making PLE a more practical and scalable technology for industry use. Innovative techniques such as employing intermittent processing and optimized pressure–temperature parameters have shown promise, enabling efficient extraction with lower energy and solvent usage while preserving compound quality.

## 4. Conclusions

This study demonstrated the potential of PLE as an efficient and sustainable method for recovering bioactive compounds from coffee silverskin, a by-product of the coffee roasting industry. The temperature and ethanol concentration were identified as critical factors in optimizing the extraction process, as revealed by a two-level factorial design. CCD and RSM further refined these parameters, revealing optimal extraction conditions of a 140 °C temperature and a 55% ethanol concentration. Experimental validation of these conditions yielded extraction outcomes that balanced multiple responses, achieving an extraction yield of 12.1%, a TEAC of 65.3 mg TE/g dry extract, a TPC of 88.4 mg GAE/g dry extract, a caffeine content of 56.7 mg/g dry extract, and a phenolic acid content of 10.6 mg CGA eq./g dry extract. Comparative analysis with alternative extraction methods highlights the advantages of the optimized PLE process, which delivers superior recovery of bioactive compounds across all measured parameters. However, the requirement for high pressure (>100 bar) and ethanol concentrations above 50% introduces challenges for industrial-scale implementation due to increased energy demands and the need for ATEX-compliant equipment. Despite these limitations, future research focusing on energy-efficient designs and solvent recycling could enhance the scalability of PLE. This study underscores the potential of PLE for the valorization of coffee silverskin, contributing to waste reduction and the development of high-value functional products while minimizing resource consumption and the environmental footprint through the use of green solvents.

## Figures and Tables

**Table 1:** Central composite design.

Factor Levels	Temperature (°C)	Ethanol Concertation (%)
+a	190	100
+1	160	80
0	108	50
−1	56	20
−a	25	0

**Table 2:** Two-level factorial design.

Temperature (°C)	EtOH (%)	Static Time (min)	S/S (g/mL)	Cycles	EY (%)	TEAC (mg TE/g Dry Extract)	TPC (mg GAE/g Dry Extract)	Caffeine (mg CAF/g Dry Extract)	Phenolic Acids (mg CGA eq./g Dry Extract)
25	100	25	1:50	0	6.23	100.9	57.9	18.8	1.00
25	100	25	1:50	0	6.29	111.4	55.4	18.5	0.93
190	100	25	1:5	2	16.9	68.7	78.7	12.8	2.34
190	100	25	1:5	2	15.3	82.1	83.3	10.8	2.17
190	0	25	1:5	0	29.9	23.7	84.3	21.9	0.99
190	0	25	1:5	0	23.3	16.9	81.5	20.4	0.29
190	0	5	1:50	0	28.7	78.8	83.1	29.3	0.44
190	0	5	1:50	0	17.2	67.4	87.7	21.5	0.70
25	0	25	1:50	2	13.2	56.9	55.2	31.9	3.44
25	0	25	1:50	2	11.7	55.4	61.4	20.4	3.29
190	100	5	1:50	2	15.9	144.7	86.2	23.4	2.45
190	100	5	1:50	2	15.1	121.9	87.6	11.5	2.37
25	0	5	1:5	2	8.29	80.4	55.1	34.2	4.02
25	0	5	1:5	2	6.82	72.9	53.1	31.5	3.87
25	100	5	1:5	0	5.66	127.3	54.3	22.9	1.20
25	100	5	1:5	0	4.50	138.2	57.3	21.2	1.08

**Table 3:** ANOVA table for each response in the two-level factorial design.

Response	Source	Sum of Squares	df	Mean Square	F	*p*-Value Prob > F
EY (%)	A-Temperature	638.1	1	638.1	55.6	<0.0001
	B-EtOH	186.1	1	186.1	16.2	0.0014
	Error	92.3	8	11.5		
TEAC (mg TE/g dry extract)	A-Temperature	2148	1	2148	31.9	0.0001
	B-EtOH	14,921	1	14,921	221.9	<0.0001
	C-Time	4541.1	1	4541	67.5	<0.0001
	D-S/S	1872.3	1	1872	27.8	0.0003
	Error	534.5	8	66.8		
TPC (mg GAE/g dry extract)	A-Temperature	31,012	1	3101	583.8	<0.0001
	D-S/S	45.8	1	45.80	8.62	0.0116
	Error	54.7	8	6.84		
Caffeine (mg CAF/g dry extract)	A-Temperature	143.4	1	143.4	9.44	0.0097
	B-EtOH	314.3	1	314.2	20.6	0.0007
	C-Time	100.9	1	100.9	6.64	0.0242
	Error	175.8	8	21.9		
Phenolic Acids (mg CGA eq./g dry extract)	A-Temperature	3.11	1	3.11	51.7	<0.0001
	B-EtOH	0.77	1	0.77	12.7	0.0039
	E-Cycles	18.7	1	18.7	311.9	<0.0001
	Error	0.32	8	0.040		

**Table 4:** Results of CCD analysis of the extraction.

Parameters				Responses		
Temperature (°C)	EtOH (%)	EY (%)	Trolox (mg TE/g Dry Extract)	TPC (mg GAE/g Dry Extract)	Caffeine (mg CAF/g Dry Extract	Phenolic Acids (mg CGA eq./g Dry Extract
108	100	7.4	73.5	30.2	42.6	2.2
56	20	11.5	63.6	57.2	30.4	7.3
25	50	18.7	38.8	57.5	35.1	6.7
160	20	19.5	13.6	27.3	20.8	3.0
190	50	26.3	27.7	58.1	36.3	8.3
160	20	18.9	13.3	24.8	24.1	2.8
108	50	6.8	74.9	126.0	59.4	12.2
190	50	29.8	35.0	63.7	30.1	6.0
160	80	20.9	84.1	50.1	33.3	6.0
56	80	9.4	31.7	38.8	34.8	6.8
56	80	10.4	29.3	62.2	40.5	5.7
56	20	10.2	42.2	40.1	26.4	4.3
108	0	12.1	34.8	33.4	33.7	3.7
108	50	7.1	77.5	103.6	68.1	13.4
108	50	6.6	66.1	101.0	57.5	13.0
108	100	11.4	74.7	41.2	44.4	2.5
108	0	9.9	29.4	25.8	25.1	2.8
160	80	21.3	73.3	59.1	33.9	5.0
108	50	6.6	75.3	120.0	65.5	12.9
25	50	15.9	45.0	79.2	42.3	9.0

**Table 5:** ANOVA table for each response in CCD.

Response	Source	Sum of Squares	df	Mean Square	F	*p*-Value Prob > F
EY (%)	Model ^1^	875.91	4	218.9	83.9	<0.0001
	Temperature	370.1	1	370.1	141.9	<0.0001
	Ethanol	18.3	1	18.3	7.02	0.0182
	Temperature ^2^	537.0	1	537.0	205.9	<0.0001
	Ethanol ^2^	18.0	1	18.0	6.94	0.0188
	Error	22.1	11	2.02		
TEAC (mg TE/g dry extract)	Model ^2^	97,212	5	1944	43.1	<0.0001
	Temperature	754.2	1	754.2	16.7	0.0011
	Ethanol	1.80	1	1.80	0.04	0.8444
	Temperature Ethanol	3847	1	3847	85.4	<0.0001
	Temperature ^2^	3187	1	3187	70.7	<0.0001
	Ethanol ^2^	987.3	1	987.3	21.9	0.0004
	Error	427.3	11	38.8		
TPC (mg GAE/g dry extract)	Model ^3^	164,903	4	4122	33.9	<0.0001
	Temperature	5112	1	5112	42.0	<0.0001
	Ethanol	15,488	1	15,488	127.3	<0.0001
	Temperature ^2^	5759	1	5759	47.3	<0.0001
	Ethanol2	15,282	1	15,282	125.7	<0.0001
	Error	1253	11	114.0		
Caffeine (mg CAF/g dry extract)	Model ^4^	32,164	4	804.1	36.4	<0.0001
	Temperature	1772	1	1772	80.4	<0.0001
	Ethanol	2249	1	2249	102.0	<0.0001
	Temperature ^2^	2002	1	2002	90.9	<0.0001
	Ethanol ^2^	1909	1	1909	86.6	<0.0001
	Error	188.5	11	17.1		
Phenolic Acids (mg chlorogenic acid/g dry extract)	Model ^5^	242.15	4	60.5	42.8	<0.0001
	Temperature	58.3	1	58.3	41.2	<0.0001
	Ethanol	224.1	1	224.1	158.4	<0.0001
	Temperature ^2^	68.2	1	68.2	48.2	<0.0001
	Ethanol ^2^	232.7	1	232.7	164.5	<0.0001
	Error	12.2	11	1.11		

R squared of each model: ^1^ R^2^ = 0.96, ^2^ R^2^ = 0.94, ^3^ R^2^ = 0.90, ^4^ R^2^ = 0.91, ^5^ R^2^ = 0.92.

**Table 6:** Predicted vs. actual responses at best extraction conditions.

Results	EY (%)	Trolox (mg TE /g Dry Extract)	TPC (mg GAE/g Dry Extract)	Caffeine (mg CAF/g Dry Extract)	Phenolic Acids (mg CGA eq./g Dry Extract)
Predicted	11.6	70.6	102.7	57.20	11.7
Actual	12.1 ± 1.53	65.3 ± 5.66	88.4 ± 7.19	56.7 ± 7.31	10.6 ± 1.46

## Data Availability

The original contributions presented in this study are included in the article. Further inquiries can be directed to the corresponding author.

## Supplementary Materials

The following supporting information can be downloaded at: https://www.mdpi.com/article/10.3390/foods14040615/s1, Figure S1: Gallic acid calibration curve used for the quantification of total phenolic content (TPC) in the samples; Figure S2: Trolox calibration curve used for the determination of antioxidant capacity (TEAC) in the samples; Table S1: UV spectra of identified compounds via HPLC-DAD.
