# Supplementary material for: Sustainable Valorization of Coffee Silverskin Waste: Pressurized Liquid Extraction of Bioactive Compounds
Source: Foods. 2025 Feb 12;14(4):615. doi: 10.3390/foods14040615 (PMC11853903; doi:10.3390/foods14040615)
Supplement: Supplementary file 1 [file foods-14-00615-s001.zip › foods-3407124-supplementary.pdf]

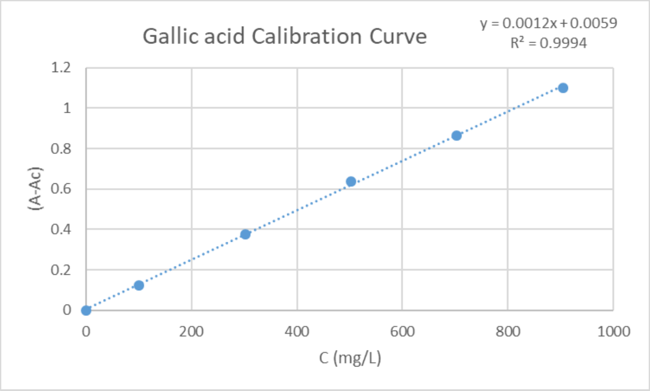

Figure S1. Gallic acid calibration curve used for the quantification of total phenolic content (TPC) in the samples.

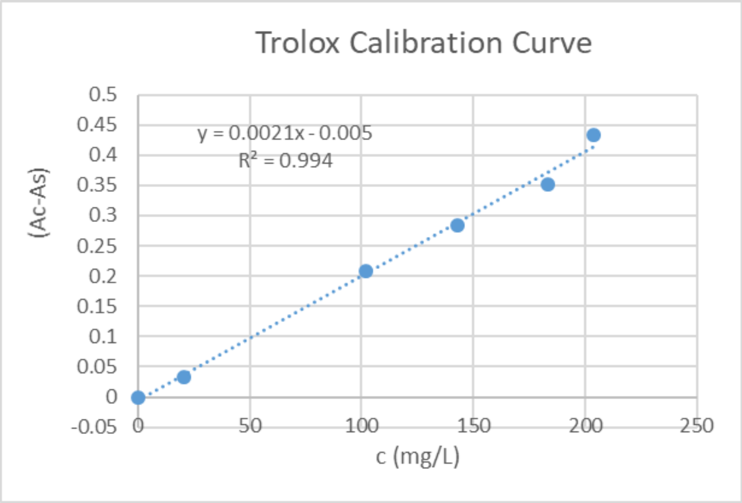

Figure S2. Trolox calibration curve used for the determination of antioxidant capacity (TEAC) in the samples.

Table S1. UV Spectra of identified compounds via HPLC-DAD.

Compound	UV Spectrum
Caffeine	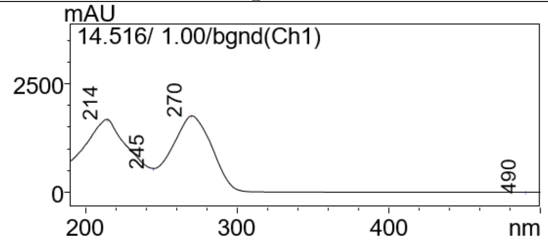
Chlorogenic acid	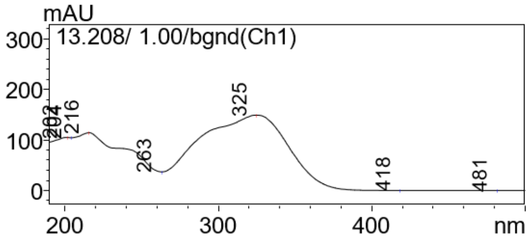

Phenolic acid 1	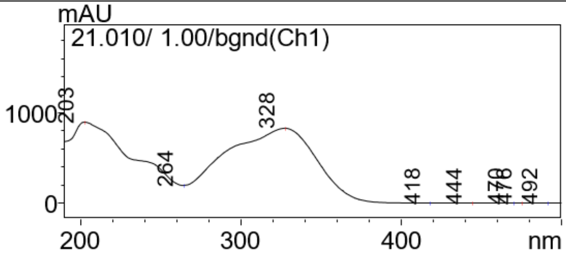
Phenolic acid 2	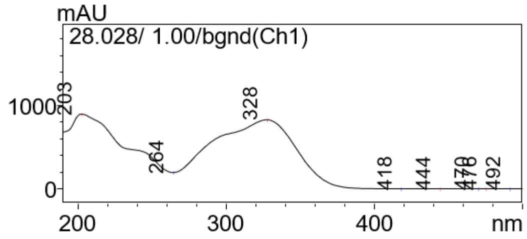
Phenolic acid 3	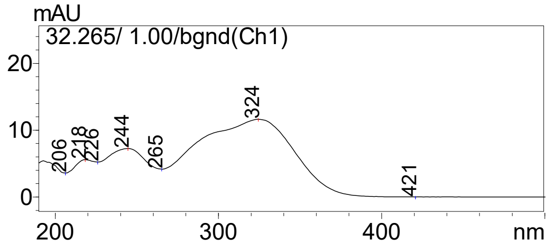
Phenolic acid 4	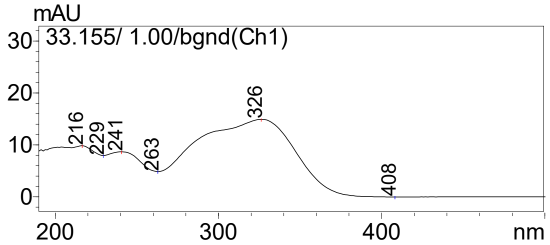
Phenolic acid 5	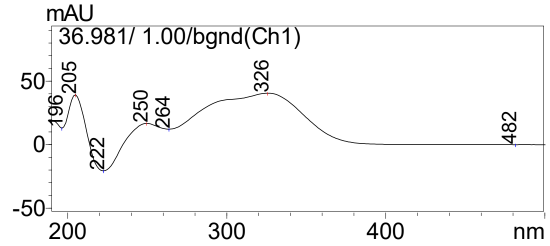
